# Inhibitory effect of K^+^ ions and influence of other ions and osmolality on the spermatozoa motility of European burbot (*Lota lota* L.)

**DOI:** 10.1371/journal.pone.0196415

**Published:** 2018-05-16

**Authors:** Katarzyna Dziewulska, Malwina Pilarska

**Affiliations:** 1 Department of General Zoology, Faculty of Biology, University of Szczecin, Szczecin, Poland; 2 Centre of Molecular Biology and Biotechnology, University of Szczecin, Szczecin, Poland; University of Hyderabad, INDIA

## Abstract

**Background:**

In fish with external fertilization, two main start-up mechanisms of the path that blocks or activates the spermatozoan motility apparatus are known. The main factor managing the path is osmolality or potassium ion. In burbot from the European and North American population, contradictory findings regarding the factors influencing the onset of spermatozoa motility were reported. The objective of the current study was to determine the effect of potassium and osmolality on the spermatozoa activation of European burbot, *Lota lota* (Actinopterygii, Gadiformes, Lotidae). Moreover, the influence of pH, as well as sodium ion concentrations on spermatozoa motility was investigated. Seven parameters characterising motility were traced by means of computer-assisted sperm analysis (CASA).

**Principal findings:**

The spermatozoa of European burbot are K^+^ ion-sensitive. A 6-mM KCl solution significantly decreased motility, and above 12-mM (50 mOsm kg^-1^) totally ceased spermatozoa movement. Sucrose and Na^+^ solutions inhibited spermatozoa movement only at concentrations > 450–480 mOsm kg^-1^. Greater differences in the percentage of motile sperm between individuals were noted in solutions containing high concentrations of chemicals triggering sperm motility. The optimum osmolality for spermatozoa motility is in the range of 100–200 mOsm kg^-1^. The burbot spermatozoa were motile over a wide range of pH values with the best activation at pH 9.

**Conclusion:**

It was demonstrated that the spermatozoa of European burbot are inhibited by K^+^ ions similarly as in North American burbot. Other electrolyte and non-electrolyte solutions inhibit spermatozoa movement only if their osmolality is greater than that of the physiological osmolality of seminal plasma. The data provided on basic knowledge of burbot spermatozoa allow to ensure appropriate conditions during artificial reproduction and scientific research.

## Introduction

External fertilization occurs in most teleostean fish. In the reproductive tract and ejaculated, milt spermatozoa remain in an inactive state until they contact with water, where they activate to swim and find eggs [1]. In fish, two main start-up mechanisms of the path that blocks or activates the spermatozoan motility apparatus are known. In the first group, osmotic pressure of the environment is the factor that triggers spermatozoa activation/inhibition. In freshwater representatives belonging to Cottidae (Scorpaeniformes), Cyprinidae (Cypriniformes), Esocidae (Esociformes), Percidae, Eleotridae, Cichlidae (Perciformes) and Clariidae, Pangasiidae (Siluriformes), solutions of osmolality lower than that of seminal plasma (<300 mOsm kg^–1^), initiates mechanism triggering the movement of spermatozoa [2–14]. By contrast, the sperm of marine teleosts begin to move when released into hypertonic water (>300 mOsm kg^–1^), and show the highest motility at approximately 1000 mOsmol kg^–1^, which is almost equivalent to seawater [15–18]. Spermatozoa of the fish from the second group, including exclusively Salmonidae (Salmoniformes), Osmeridae, Plecoglossidae (Osmeriformes) and Acipenseridae, Polyodontidae (Acipenseriformes), are managed by K^+^ ions. The presence of K^+^ ions in the external environment at the appropriate concentration inhibits spermatozoa motility. The initiation of motility is triggered by a removal in the K^+^ concentration in the external environment [15,19–31]. However, presence of some ions overcome the inhibitory effect of the K+ ions. Mostly divalent ions, such as calcium, magnesium, manganese, as well as sodium and lithium ions antagonise the K^+^ suppression [25, 32–41]. The acting threshold of the ions concentration is species specific [40].

In both groups of fish characterized by different factors triggering the activation, flux of the K^+^ ions through plasma membrane plays a key role in the maintenance of membrane potential. In the first group of fish with osmolality-dependent signaling systems, osmotic shock is converted into change in the intracellular K^+^ concentration, which induces transduction of the signal to flagellar axoneme. In freshwater fish hypotonic signal induces swelling and efflux of K^+^ ion resulting membrane hyperpolarization [2,42,43]. In the group of fish with K^+^ ion-sensitive spermatozoa, a decrease in the external K^+^ concentration induces K^+^ efflux from the cell and membrane hyperpolarization [22,32–34,44–46]. In salmonids addition of specific voltage-dependant K^+^ channels blockers inhibit sperm motility [44–46] while in case of osmolality-dependant spermatozoa of puffer their motility is not influenced by potassium channel blockers [47] or decrease duration of motility in carp [48]. In carp potassium ionophore did not initiate sperm motility and did not affect sperm motility when spermatozoa are motile [47]. In salmonids not only the K^+^ channel, but also the Ca^2+^, H^+^ and Na^+^ channels are involved in membrane hyperpolarization [35,41]. Membrane hyperpolarization triggers the cascade of events leading to spermatozoa activation through cyclic AMP- dependent phosphorylation of axonemal proteins or Ca^2+^-dependent calmodulin phosphorylation [14,30,34,35,43,49–56].

The burbot (*Lota lota*) is an endangered species in many parts of world. Artificial insemination and restocking of offspring is a necessity to preservation of the species [57–61]. In burbot, contradictory findings regarding the factor triggering the onset of spermatozoa activation were drawn. In European burbot, Lahnsteiner et al. [62] by testing spermatozoa activation with solutions of NaCl, glucose, sucrose ranged in osmolality 25–400 mOsm kg^-1^, pH range from 6.0 to 9.0 and solutions containing a mixture of NaCl and 5–30 mM KCl of final osmolality 100–400 mOsm kg^-1^ reviled that the burbot spermatozoa are not K^+^-sensitive and osmolality is the factor governing spermatozoa motility activation/inactivation. In North American burbot, Zuccarelli et al. [63] stored milt in Hank’s incubation solution containing 120 mM NaCl, 0.25 mM Na_2_HPO_4_, 0.44 mM K_2_HPO_4_, 1.3 mM CaCl_2_, 1 mM MgSO_4_, at pH 8.5 for 1 h then assessed motility in tap water. In individual experiments Ca^2+^ ion was discarded from the solution or greater concentrations of ions (Ca^2+^, Na^+^, K^+^) was added to the basic solution. Moreover the influence of pH and CO_2_ was tested. An inhibitory effect of K^+^, osmolality and pH on spermatozoa motility was designed by Zuccarelli et al. [63]. The contradictory findings regarding the factor suppressing the spermatozoa of burbot derived from two regions was congruent with the historically described subspecies, *L*. *lota lota* and *L*. *lota maculosa* and the hypothesis of formation of two distinct phylogroups within the *Lota* genus [64,65].

In view of different factors influencing the onset of spermatozoa motility were reported in the two different groups of burbot the objective of the current study was to investigate the effect of potassium and osmolality as simple factors on the gamete of European burbot. Moreover the influence of pH and sodium ions on the spermatozoa activation and motility parameters was examined. The composition of seminal plasma was determined and compared with that of other taxa. Disclosure of the matter is important for the basic knowledge on sperm biology of burbot. This knowledge is needed to ensure appropriate conditions during artificial spawning and scientific research, to develop activation, inhibiting and storage solutions appropriate to the species.

## Materials and methods

### Sperm collection

European burbot, *Lota lota* L. (Lotidae, Gadiformes) individuals of a size exceeding the protected size (40–58 cm long) were caught in the Oder River (NW Poland) by employees of the Polish Angling Association (PAA) in Szczecin and transported to the PAA hatchery in Goleniów. There is no protection period for burbot in that area. Fishing license for the District of the PAA was issued by the Regional Board of Water Management in Szczecin. No special field sampling permits for sites locations, e.g. for national park or other protected area, were necessary. The PAA does not need any permits or permissions for keeping and breeding fish in a hatchery. The fish were kept in the hatchery for a short period until spawning in a flow-through tank supplied with river water under a natural photoperiod. The fish density in the tank (20 kg m^-3^) was in the appropriate range for keeping this species. The fish were not fed for one week. Samples of semen for the study were obtained in the PAA hatchery during the activities related to the controlled breeding of the species in January. The milt was stripped by hand by the PAA employees into plastic containers, and care was taken to avoid contamination by urine and faeces. No anaesthesia was used (as permitted by law), and the management aimed to minimise pain and suffering of the fish. The males were not stimulated hormonally. The experimental procedures conducted on semen do not require permission from the Ethics Committee for Animal Experimentation according to Polish law. Milt was transported to laboratory in plastic containers on ice (2–4°C) for less than 1 h until analysis began.

### Computer assisted sperm analysis (CASA)

Spermatozoan motility was evaluated with an automated system, the Sperm Class Analyzer (SCA) v. 4.0.0. by Microptic S.L., Barcelona, Spain. Activation was triggered with a 200-300-fold dilution in the activating solution (30 mM NaCl, at 4°C) with the addition of 0.1% bovine serum albumin (BSA). Milt (1–2 μL) was added to 300 μL of the activating solution in a 1.5 mL polyethylene Eppendorf tube. The temperature of the solution in the tube was maintained using a cooling block (FINEPCR, Korea), at 4°C. After intense stirring of the tube contents for 2–3 seconds, 1.2 μL of this dilution was immediately placed into a well of a 12-well multi-test glass slide (MP Biomedicals LLC, Germany) and covered with a coverslip. The microscope table was equipped with a cooling device (Semic Bioelektronika, Kraków, Poland) set at 4°C. Spermatozoan movement was monitored using a camera (Basler A312fc, with sensor type CCD ½”) at 50 Hz mounted on a negative phase contrast Nikon Eclipse 50i microscope linked with a CASA device, at 250 x magnification. Settings in the SCA were particle area from 1 to 30 μm^2^, velocity of average path (VAP) points of 5, brightness 128 and contrast 128. Half-second films (25 frames) were recorded at 10 s and every 20 seconds thereafter until motility completely ceased. Activation and film recording were repeated three times. Mean values of the parameters for each individual were obtained from the average of three activations of spermatozoa. Spermatozoan concentration was assessed in a Bürker chamber counting by the SCA at a dilution of 4000x with 0.8% NaCl. Twice for each male spermatozoa were counted in 10 squares after which the mean of 10 fields and the average of the two values was computed, giving the spermatozoan concentration.

Milt from selected four fish was used to test influence of pH, cation and sugar concentrations in the activation buffer on spermatozoan motility. Seven parameters characterising motility were chosen for analysis: 1. MOT—percentage of motile spermatozoa (where the criterion of motility was an average path velocity >20 μm s^-1^), 2. VCL—curvilinear velocity (μm s^-1^), 3. VAP—average path velocity (μm s^-1^), 4. VSL–straightline velocity (μm s^-1^), 5. LIN—linearity (VSL/VCL x 100), 6. ALH—amplitude of lateral head displacement (μm) i.e. the magnitude of lateral movement of the spermatozoan head with respect to its average track, 7. BCF—beat cross frequency (Hz) i.e. the track crossing frequencies or the average frequency at which a sperm cell’s curvilinear track crossed its average track and motility duration, the time interval from activation to cessation of motility of all spermatozoa.

### Effects of pH on spermatozoan motility

The effects of pH on spermatozoan motility were tested in the range of pH 4–13. Percentage of motile spermatozoa, motility parameters and duration of motility were measured using computer-assisted analysis as described above. A solution of 20 mM MES–NaOH buffer was used to obtain pH ranges 4–7, 20 mM Tris–HCl buffer to get pH ranges 8–10 and 20 mM Tris–NaOH to get pH ranges 11–13. All buffers were supplemented with 0.1% BSA. The albumin content added to prepared solution is similar to the concentration of proteins in seminal plasma of burbot [62]. Osmotic pressure of the buffer was measured using a Trident osmometer 800 cl and ranged from 25 to 30 mOsm kg^-1^. Spermatozoa were activated at the dilution ratio about 1:300 with each solution (4°C).

### Effects of KCl, NaCl concentrations on spermatozoan motility

The effects of ion concentrations on sperm motility were tested in ion solutions dissolved in 20 mM Tris–HCl buffer containing 0.1% BSA (pH 9). The buffer was supplemented with:

1. 0, 1, 2, 4, 6, 8, 10, 12, 14 mM KCl. Osmolalities of the buffers were 25, 27, 29, 33, 37, 41, 45, 49, 53 mOsm kg^-1^, respectively.
2. 0, 30, 60, 90, 120, 150, 180, 210, 240, 270 mM NaCl. Osmolalities of the buffers were 25, 85, 140, 195, 250, 300, 350, 400, 450, 500 mOsm kg^-1^, respectively.

Spermatozoa were activated at the dilution ratio about 1:300 with each solution (4°C).

### Effects of sugar concentrations (osmolality) on spermatozoan motility

The effects of sucrose and glucose concentrations on sperm motility were tested in sugar solutions dissolved in 20 mM Tris–HCL buffer containing 0.1% BSA (pH 9). The buffer was supplemented with:

1. 0, 80, 160, 240, 320, 400, 440, 480 mM sucrose. Osmolalities of the buffers were 25, 110, 200, 290, 380, 480, 530, 580 mOsm kg^-1^, respectively.
2. 0, 80, 160, 240, 320, 400, 440, 480 mM glucose. Osmolalities of the buffers were 25, 100, 180, 260, 340, 420, 460, 520 mOsm kg^-1^, respectively.

Spermatozoa were activated at the dilution ratio about 1:300 with each solution (4°C).

### Determining seminal plasma indices

The milt was centrifuged to obtain the seminal plasma at 8,000 *g* for 10 min at 4°C, the supernatant was recentrifuged under the same conditions. In the seminal plasma the concentrations of Na^+^, K^+^_,_ Cl^-^ Ca^2+,^ ions and pH were determined by ion-selective electrodes (BM ISE Electrolyte Analyzer, BioMaxima). The concentrations of Mg^2+^ were determined by colorimetric methods (Architect cSystem, Abbott). Osmolality were determined using a Trident 800 cl osmometer.

### Statistical analysis

One-way repeated measures ANOVA was used to test the effect of ions and sugars concentration or pH value upon the duration of spermatozoan movement. Two-way repeated measures ANOVA was used to test the effects of (1) ions and sugars concentration or pH value and (2) time post-activation upon other measured motility parameters. Because spermatozoa became less motile with time, two-way ANOVAs were performed on the data subsets covering those time and ions and sugars concentration/pH ranges for which sufficient numbers of motile spermatozoa were observed. When a significant interaction effect was found in a two-way analysis, separate one-way ANOVAs were run for each post-activation time to check for time-specific effects of ions and sugars concentration or pH value. Moreover, separate one-way ANOVAs were used to check for differences among various post-activation times for particular ions and sugars concentrations and pH values. Tukey’s test was used for all subsequent post-hoc comparisons. For simplicity, only the differences within R1 factor for particular post-activation times were considered in the cases of significant interactions. All statistical procedures were performed with Statistica 12.0 software, and the results were regarded as statistically significant at a level of 0.05.

## Results

### Milt and seminal plasma composition

The spermatozoa concentration in burbot milt was 39.2±12.2 x 10^9^ mL^-1^. Osmolality of seminal plasma was 261.3± 14.1 mOsm kg^-1^. Ions concentration and pH in the seminal plasma are presented in Table 1 (n = 6).

**Table 1:** Ionic composition of seminal plasma of fish with K^+^ sensitive spermatozoa.

Species	pH	Osmolality	Na^+^	K^+^	Ca^2+^	Mg^2+^	Cl^-^	Author
		[mOsm kg^-1^]	[mM L^-1^]	[mM L^-1^]	[mM L^-1^]	[mM L^-1^]	[mM L^-1^]	
*Lota lota*	8.2	261.3	102.7	18.9	2.0	1.2	77.7	Present paper
*Lota lota*	8.47	290.1	139.9	11.6	0.20			[62]
*Lota lota maculosa*	8.55	253	110	23.5	3.5			[63]
*Oncorhynchus mykiss*	7.99	316	128.9	32.5	2.7	1.02	143	[66]
*Oncorhynchus mykiss*	8.11	290	116.5	20.2	0.8			[67]
*Oncorhynchus masou*		301	126	10.8	2.1	1.0	130	[19]
*Oncorhynchus keta*		332	141	66.0	0.95	3.6	133.7	[68]
*Oncorhynchus tshawytscha*		265	110	37.3	0.65	1.15	109	[69]
*Salmo salar*	8.25	232	103.0	22.0	1.3	0.9		[70]
*Salmo trutta* m. *trutta*	8.00	272.0	127.3	24.8	1.68	1.22	84.5	[71]
*Salmo trutta caspius*			159.2	33.7	1.7	1	133.0	[72]
*Salvelinus fontinalis*	7.96	248.5	136.6	30.5	0.40	0.69	135.6	[71]
*Acipenser persicus*		82.6	62.4	6.9	0.79	0.52	21.1	[73]
*Acipenser fluvescens*			25.6	5.8	0.16	0.20	5.4	[24]

### Effect of pH on spermatozoa motility

**Fig 1:**
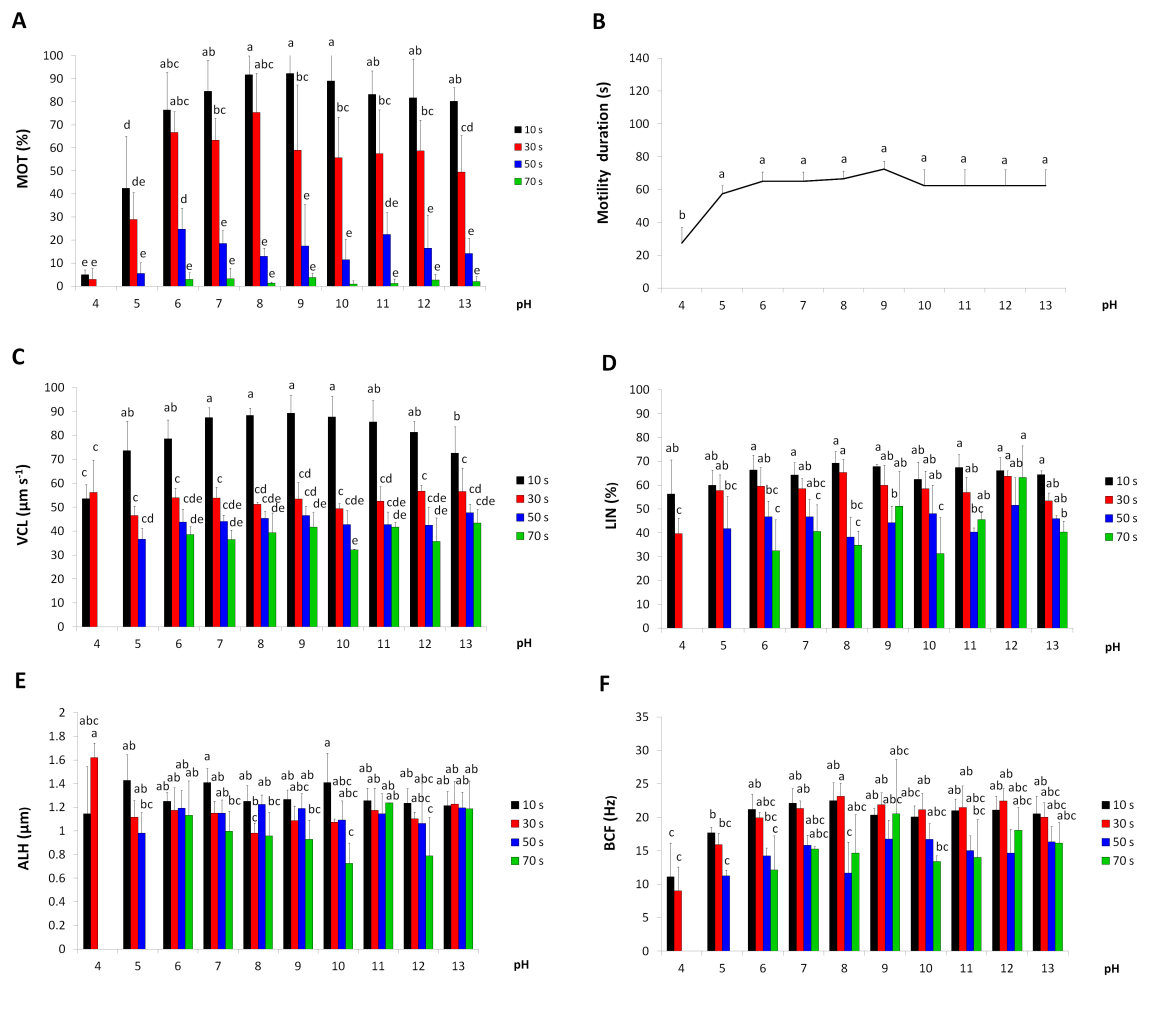
The effects of pH on European burbot spermatozoa motility at particular times after activation. (A) percentage of motile spermatozoa (MOT). (B) duration of motility. (C) curvilinear velocity (VCL). (D) linearity (LIN). (E) amplitude of lateral head displacement (ALH). (F) beat cross frequency (BFC). Values marked with the same letter are not significantly different from one another (P>0.05). Two-way or one-way ANOVAs and then Tukey tests were used for the post-hoc comparison. Mean value ± SEM.

Motility (MOT) was significantly influenced by the pH value and time post-activation. At activation, a significantly lower value of MOT was noted only at pH 4 and 5, while in the pH range of 6 to13, motility was similar (Fig 1A).

Motility duration was significantly affected by the pH value. At pH 4, the duration of movement was significantly lower than those observed at higher pH values. In the pH range of 5–13, the duration of motility was similar (Fig 1B).

Significant pH value x post-activation time interactions were found for three velocities: curvilinear (VCL), average path (VAP) and straightline (VSL). One-way ANOVAs revealed that significant differences in velocities between various pH values occurred at 10 s post-activation, but not at subsequent post-activation intervals. At 10 s post-activation, lower values of VCL were triggered at pH 4 and 13, VAP at pH 4–6 and 13, while VSL at pH 4–5. The velocities decreased across the motility phase, a significant drop occurred between 10 and 15 s post-activation (Fig 1C).

Linearity (LIN) was not influenced by the pH value and activation time (Fig 1D).

A significant pH value x post-activation time interaction was found in the ALH analysis. One-way ANOVAs revealed that significant differences in ALH between various pH values occurred only at 30 s post-activation. At 30 s post-activation, the highest values of ALH were noted at pH 4, being significantly higher than those noted at pH 8 (Fig 1E).

The values of BCF were influenced by pH. The lowest values of the parameter were noted at acidic pH, particularly at pH 4 (Fig 1F).

### Effect of KCl concentration on spermatozoa motility

**Fig 2:**
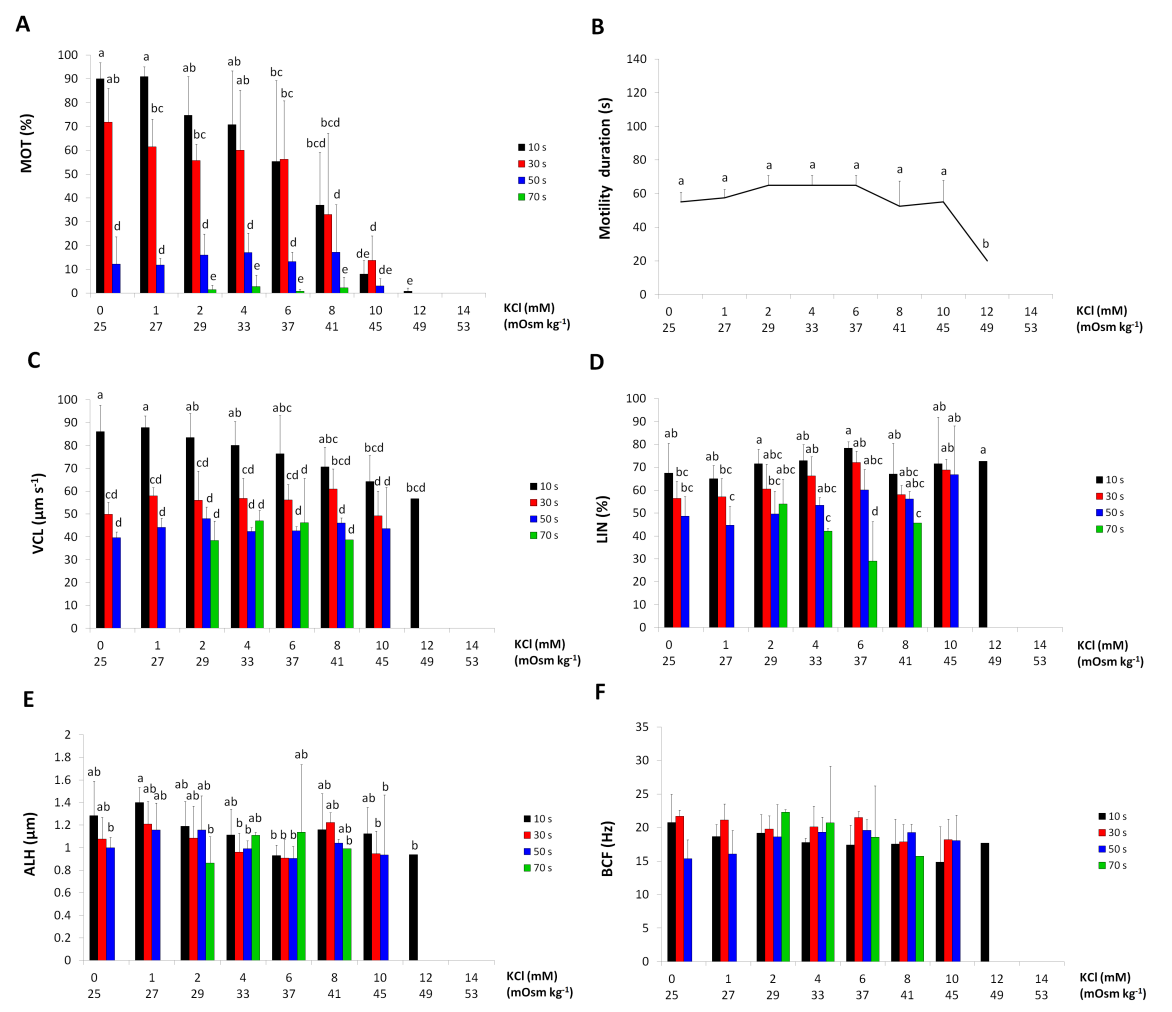
The effects of KCl concentrations on European burbot spermatozoa motility at particular times after activation. (A) percentage of motile spermatozoa (MOT). (B) duration of motility. (C) curvilinear velocity (VCL). (D) linearity (LIN). (E) amplitude of lateral head displacement (ALH). (F) beat cross frequency (BFC). Values marked with the same letter are not significantly different from one another (P>0.05). Two-way or one-way ANOVAs and then Tukey tests were used for the post-hoc comparison. Mean value ± SEM.

A significant KCl concentration x post-activation time interaction was found for MOT. One-way ANOVAs for separate post-activation times revealed that a significant effect of KCl concentration on MOT was observed at 10 s and 30 s post-activation time, but not at 50 s and 70 s. The percentage of motile spermatozoa decreased with increasing KCl concentration at the first two measurement times. A significant decrease was observed at 10 s post-activation with 6 mM KCl and at 30 s post-activation with 10 mM KCl. Total inhibition of spermatozoa motility was observed with KCl over 12 mM (49 mOsm kg^-1^). Greater differences in MOT between individuals were obtained in solutions with high concentrations of KCl triggering spermatozoa activation (Fig 2A).

Motility duration was stable across the KCl concentrations with a significant drop only at 12 mM KCl, the highest concentration triggering motility (Fig 2B).

Only for VAP, a significant KCl concentration x post-activation time interaction was found. VCL and VSL were influenced by post-activation time—both values decreased as motility progressed (Fig 2C). One-way ANOVAs revealed that significant differences in VAP between various KCl concentrations occurred at 10 s post-activation. At 10 s post-activation, VAP was stable in the range 0–8 mM KCl, while decreased at the solution concentrations of 10 mM and 12 mM.

The LIN and ALH values depended only on post-activation time. The values decreased or were similar as time passed after activation (Fig 2D and 2E).

BCF was not influenced by any of the factors, and its values differed modestly across the KCl concentrations (Fig 2F).

### Effect of sucrose concentration (osmolality of non-electrolyte solution) on spermatozoa motility

**Fig 3:**
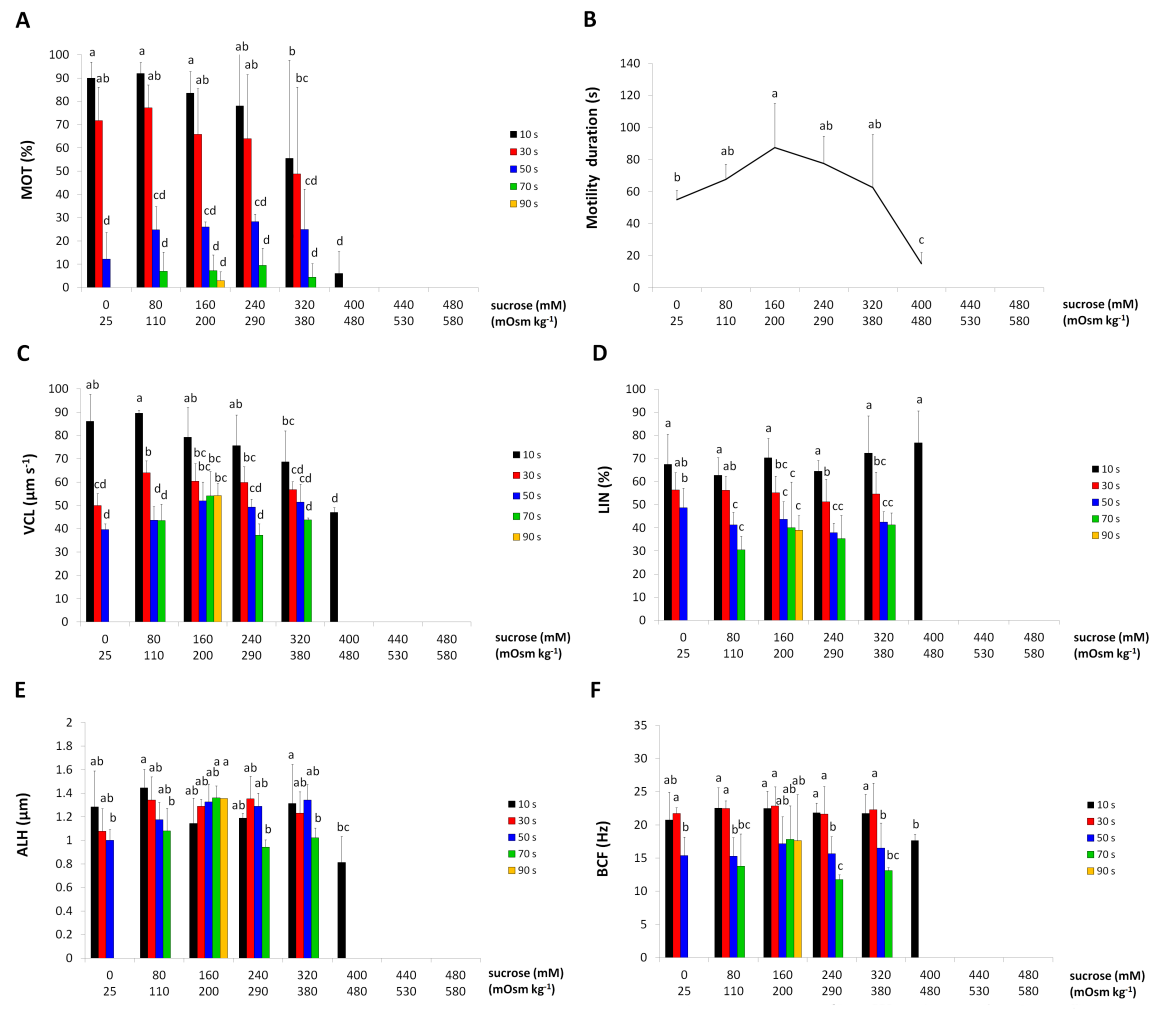
The effects of sucrose concentrations on European burbot spermatozoa motility at particular times after activation. (A) percentage of motile spermatozoa (MOT). (B) duration of motility. (C) curvilinear velocity (VCL). (D) linearity (LIN). (E) amplitude of lateral head displacement (ALH). (F) beat cross frequency (BFC). Values marked with the same letter are not significantly different from one another (P>0.05). Two-way or one-way ANOVAs and then Tukey tests were used for the post-hoc comparison. Mean value ± SEM.

A significant sucrose concentration x post-activation time interaction was found for MOT. One-way ANOVAs run for particular post-activation times revealed that a significant effect of sucrose concentration on MOT was observed only at 10 s post-activation time, but not at 30 s, 50 s and 70 s. A significant decrease in MOT at that interval occurred with 320 mM sucrose (380 mOsm kg^-1^). Total inhibition of spermatozoan motility was observed with sucrose over 400 mM (480 mOsm kg^-1^). Greater differences in MOT between individuals were obtained in solutions of high concentrations of sucrose triggering spermatozoa activation (Fig 3A).

Motility duration was influenced by the sucrose concentration. The parameter increased from 55.0 ± 5.7 s in buffered water to 87.5 s in 160 mM sucrose (200 mOsm kg^-1^), and was reduced at higher sucrose concentrations (Fig 3B).

A highly significant interaction was found between post-activation time and the sucrose concentration for VCL and VAP. VSL depended only on post-activation time, decreasing with motility. One-way ANOVAs revealed that significant differences between various sucrose concentrations occurred for VCL at 10 s, 30 s and 50 s post-activation. At 10 s post-activation, an increase in the sucrose concentration led to a gradual decrease in VCL, significant with a sucrose concentration of 320 mM (380 mOsm kg^-1^). At 30 s post-activation, spermatozoa maintained the highest value of VCL in 110–280 mOsm kg^-1^ sucrose solutions, while at 50 s post-activation, the parameter was the greatest in 200–300 mOsm kg^-1^ media (Fig 3C). One-way ANOVAs revealed that significant differences in VAP between various sucrose concentrations occurred only at 10 s. A significant decrease in VAP at activation was noted only with 400 mM sucrose (480 mOsm kg^-1^), the highest concentration triggering motility. At other measurement times, the VAP values were similar with all sucrose concentrations

The LIN and BCF values depended only on post-activation time. The LIN values decreased with time. BCF in the first phase of motility was similar, then decreased toward the end phase of motility (Fig 3D and 3E).

A significant sucrose concentration x post-activation time interaction occurred for ALH. A significant effect of the sucrose concentration on ALH occurred only at 70 s, with ALH at 200 mOsm kg^-1^ being greater than the values observed with the other sucrose concentrations (Fig 3F).

The effect of glucose solutions on the burbot spermatozoa motility was similar to that of sucrose.

### Effect of NaCl concentration on spermatozoa motility

**Fig 4:**
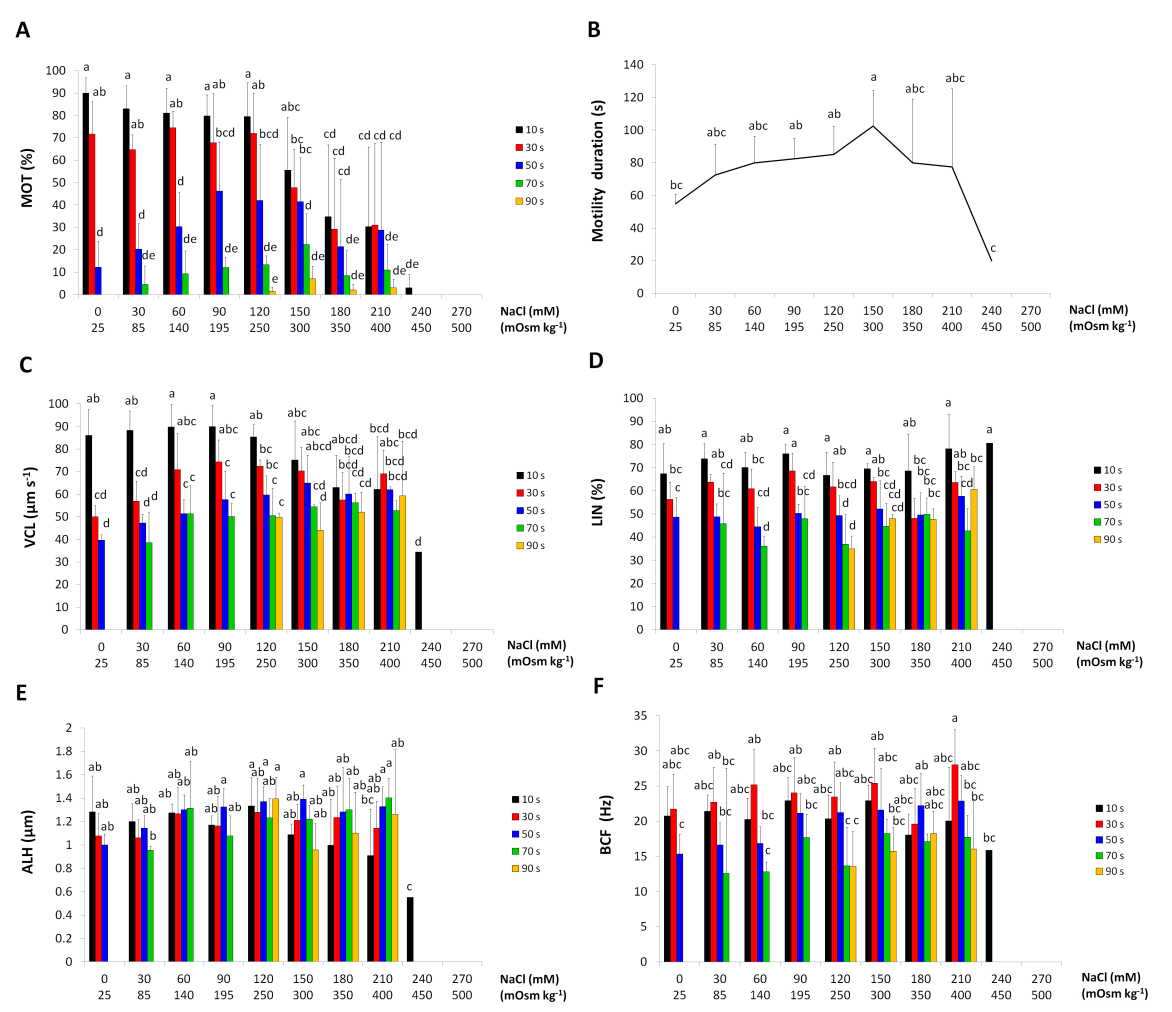
The effects of NaCl concentrations on European burbot spermatozoa motility at particular times after activation. (A) percentage of motile spermatozoa (MOT). (B) duration of motility. (C) curvilinear velocity (VCL). (D) linearity (LIN). (E) amplitude of lateral head displacement (ALH). (F) beat cross frequency (BFC). Values marked with the same letter are not significantly different from one another (P>0.05). Two-way or one-way ANOVAs and then Tukey tests were used for the post-hoc comparison. Mean value ± SEM.

A highly significant interaction was found between the NaCl concentration and post-activation time for MOT. One-way ANOVAs showed that the effect of the NaCl concentration on MOT was significant at 10 s and 30 s post-activation, but not in the later phase of motility. At 10 s and 30 s post-activation, the percentage of motile spermatozoa remained similar at the NaCl concentrations up to 150 mM (300 mOsm kg^-1^). At higher concentrations, MOT gradually decreased, and over 240 mM (450 mOsm kg^-1^) NaCl, motility was totally eliminated. Greater differences in MOT between individuals were obtained in solutions of high concentrations of NaCl triggering spermatozoa activation (Fig 4A).

In contrast to the effects of KCl and CaCl_2_, but similarly to the effects of sucrose and glucose, increasing concentration of NaCl increased the duration of motility up to 102.5 ± 23.2 s with 150 mM NaCl (300 mOsm kg^-1^), then decreased with an increasing concentration of NaCl (Fig 4B).

A significant NaCl concentration x post-activation time interaction was observed for VCL, VAP and VSL. One-way ANOVAs revealed that a significant effect of the NaCl concentration on VCL occurred from 10 s to 70 s intervals, while for VAP and VSL—at 10 s and 30 s post-activation. At 10 s post-activation, the VCL values did not differ significantly in the prepared NaCl solutions up to 180 mM NaCl (ranged from 63.0 ± 14.3 μm s^-1^ to 90.0 ± 9.3 μm s^-1^), but declined at higher NaCl concentrations. At 30 s and 50 s post-activation, VCL increased with increasing NaCl concentration up to 90 mM and 150 mM, respectively, and then slightly decreased (Fig 4C). At 70 s post-activation, the parameter increased, being higher with NaCl concentrations of over 60 mM NaCl than with 30 mM NaCl. At 10 s post-activation, VAP and VSL slightly increased, and then decreased significantly with 180 mM NaCl concentration. At 30 s, the velocities increased with increasing NaCl concentration up to 90 mM NaCl, then decreased in higher NaCl concentrations. At 50 s, 70 s and 90 s post-activation, the values of VAP and VSL in all sodium chloride solutions did not differ significantly.

LIN and BCF depended only on post-activation time. LIN decreased in the course of the motility phase, while BCF increased from 10 s to 30 s, and then decreased, with the exception of the 180 mM solution in which the parameter increased until 50 s post-activation and then decreased (Fig 4D and 4E).

A significant NaCl concentration x post-activation time interaction was found for ALH. However, one-way ANOVAs showed a significant effect of the NaCl concentration on ALH at 10 s post-activation. At activation, the ALH value was lower with higher NaCl concentrations, but a significant drop was observed in the 210 mM NaCl solution (Fig 4F).

The effect of Na^+^ ion on spermatozoa activation was similar to the effect of solution containing sucrose. Considering the value of percentage of motile spermatozoa, velocity and time of motility duration optimum osmolality of the activating solution for burbot spermatozoa activation is in the range of 100–200 mOsm kg^-1^.

## Discussion

In fish, two main start-up mechanisms of the path that blocks or activates the spermatozoan motility apparatus are known. The main factors controlling spermatozoa activation are osmolality or potassium ions [15,17,20,40]. Until now in two papers published about the burbot spermatozoa activation mechanisms, other factors inhibiting the gamete have been indicated. In European burbot osmolality was indicated as the inhibiting factor [62] while in North American burbot, potassium ion, osmolality and possibly pH were listed as factors maintaining spermatozoa in an immotile state [63]. In both studies spermatozoa was treated with solutions containing mixture of ions. In the case of European burbot, Lahnsteiner et al. [62] testing influence of K^+^ ion used activating solution containing KCl in a concentration of 5 (10)– 30 (40) mM (different range is mentioned in Material and methods and Results sections) with addition of imprecise content of NaCl. Final osmolality of the solution ranged between 100–400 mOsm kg^-1^. Analysis were performed in the Makler chamber. Authors obtained similar motility parameters in NaCl solutions as in solutions containing potassium ions of same osmolality, therefore effect of potassium was omitted. In presented studies on European burbot spermatozoa potassium ion was used as a simple factor to test its inhibitory effect. In our studies inhibitory effect of K^+^ ion on spermatozoa motility was indisputable. Not getting inhibitory effect of potassium on burbot spermatozoa by Lahnsteiner et al. [62] could be caused by antagonize effect of sodium to potassium ion mixed together in the activating solution. The antagonizing effect of sodium ion to potassium ion was described in rainbow trout [19,32] but this requires verification in burbot. Inhibitory effect of K^+^ usually reverse divalent ions. The effect of Na^+^ is weaker than this of divalent ion [32,38,39]. Also spontaneous activation of spermatozoa could affect the results but the phenomenon was known by the authors. In their studies spontaneous activation of spermatozoa above 5°C was discovered therefore experiment was performed at 4°C. Zuccarelli et al. [63] did not note spontaneous activation until 10°C while Dadras et al. [74] did not observe that phenomenon up to 20°C. In higher temperature spontaneous motility was seen whereas at 30°C most of spermatozoa were motile [74,75]. Another reason for the result obtained by Lahnsteiner et al. [62] could be lack of dryness or cleanness of the chamber used during analysis.

The potassium ions is the primary factor that inhibits spermatozoa in fish considered as a primitive group of teleosts that migrate from marine water to freshwater to reproduce, retaining their resistance to hypoosmolality [19]. The burbot is the only member of Lotidae family which lives in freshwater [76]. The concentration of K^+^ that exerts an inhibitory effect on spermatozoa is different in every species. In the studied *L*. *lota* (Lotidae, Gadiformes), a total suppression by K^+^ of all spermatozoa was obtained at 12 mM KCl. Zuccarelli et al. [63] in North American burbot the best results of milt storage noted when solution containing divalent ions of osmolality >200 mOsm kg^-1^ was supplemented with 12.5 mM KCl. In *Osmerus eperlanus* (Osmeridae, Osmeriformes), a concentration of 10 mM clearly reduced the percentage of motile spermatozoa [26]. In *Coregonus albula* and *Oncorhynchus spp*. (Salmonidae, Salmoniformes), this effect was obtained at a concentration of around 8–10 mM [15,28,35,37], whereas in the *Salmo* genus the effective KCl concentration is lower than 6–8 mM [31,Dziewulska unpublished data]. In Acipenseriformes, fish belong to Chondrostei in total, inhibition was induced by very low K^+^ concentrations below or around 2 mM [23–25,29,30]. This effect could fully reverse the Ca^2+^ ion [23,25] for *Acipenser ruthenus* at a Ca^2+^: K^+^ ratio of 0.25 [30].

In the second group of fish with osmolality-dependant spermatozoa, the K^+^ ions do not have an inhibitory effect and, as other ions solutions act through osmotic pressure. Although solution supplemented with potassium, prolonged motility duration in *E*. *masquinongy* [4]. In *Perca fluviatilis* 0.25 mM K^+^ increased velocity [77]. A total suppression of spermatozoa in this group of fish by electrolyte and non-electrolyte solutions is obtained at osmolality lower or roughly equal to that of seminal plasma. The acting osmolality depends on the species and, to some extent, on the chemical compound. In Cyprinidae (Cypriniformes), the immobilizing effect is seen with a solution of osmolality over 250 mOsm kg^-1^ in *Cyprinus carpio* [78] and in *Puntius javanicus* [9]. More than 285 mOsm kg^-1^ is needed to obtain this effect in *Vimba vimba* [79], approx. 300 mOsm kg^-1^ in *Clinostomus elongates* [14], 330–350 mOsm kg^-1^ in *Barbus barbus* [11]. In Esocidae (Esociformes), the effect is observed at 300–375 mOsm kg^-1^ in *Esox* [4,12]. In Claridae (Siluriformes)–at 250–300 mOsm kg^-1^ in *Clarias* [7,9]. In Percidae (Perciformes)–at 300 mOsm kg^-1^ in *P*. *fluviatilis* [3,10], in *Oxyeleotris marmorata* (Eleotridae), the effect for non-electrolyte solution is obtained at 250 mOsm kg^-1^, for KCl solution at 150 mOsm kg^-1^, while for NaCl solutions the effective osmolality is intermediate [9].

In the fish from the group of K^+^ sensitive spermatozoa, solutions of osmolality significantly exceeding the osmolality of seminal plasma totally suppressed motility. In *L*. *lota* investigated in presented work, the threshold of osmolality of NaCl, sucrose, glucose solutions was above 450–480 mOsm kg^-1^. In European burbot studied by Lahnsteiner et al. [62] osmolality >400 mOsm kg^-1^ was inhibitory. In other species of fish with K^+^ sensitive spermatozoa such as *O*. *mykiss* non-electrolyte and NaCl solutions at 340 and 450 mOsm kg^-1^, respectively, suppressed motility, while in *O*. *keta*, motility was blocked by solutions of 450 and 500 mOsm kg^-1^, respectively [15,19]. In *C*. *albula*, NaCl concentrations as low as 60 mM decreased the percentage of sperm motility. This phenomenon distinguishes vendace sperm from that of other salmonids [28]. In sturgeon and paddlefish, the osmolality of seminal plasma is much lower than 100 mOsm kg^-1^. For sturgeon, the osmolality suppressing spermatozoa is as high as 175 mOsm kg^-1^ [29,30].

High osmolality suppressed motility while a specific osmolality of solution results in prolongation of time of movement maintaining high velocity and percentage of motile spermatozoa in the condition. The optimum osmolality for spermatozoa movement in the studied *L*. *lota* was 100–200 mOsm kg^-1^, which is similar range for salmonids [15,19,27,31]. In Acipenseriformes the optimal movement of spermatozoa is detected at an osmolality of 50–75 mOsm kg^-1^ [29,30]. In the fish of the second group of osmolality-regulated spermatozoa activation, the optimum osmolality for motility is close to the fish with K^+^-dependant spermatozoa exclude Acipenseriformes. In *C*. *elongates* the range is 105–225 mOsm kg^-1^ [14], 200 mOsm kg^-1^ for *V*. *vimba* [79], 215–230 mOsm kg^-1^ for *B*. *barbus* [11], 100–150 mOsm kg^-1^ for *P*. *fluviatilis* [3,10]; 70–333 mOsm kg^-1^ for freshwater *O*. *mossambicus* [5], 125–235 mOsm kg^-1^ for *E*. *lucius* [12], 200 mOsm kg^-1^ for *C*. *macrocephalus* [7].

Apart from two main aforementioned factors affecting activation and spermatozoa motility, pH, CO_2_, temperature, ovarian fluid, peptides from egg coat and other factors also play an important role in their regulation [17,40,80,81]. External pH induces a change in the internal pH and influences membrane polarization [22]. Subsequently, the factor affects spermatozoa activation, motility pattern and fertilization success. Alkaline medium is more suitable for spermatozoa activation, although the sensitivity of gametes to the factor is species-specific [66, 80,82,83]. The spermatozoa of the studied *L*. *lota* were motile in a wide spectrum of external pH. High motility was occurred between pH 7 and pH 12, with the best motility characteristics triggered at pH 9. A negative effect on motility was seen in acidified solutions. Zuccarelli et al. [63] also determine low sensitivity to pH by North American burbot spermatozoa. Motility was significantly inhibited only in water of pH 6.5. Gametes of other fish except for *C*. *microcephalus* [7], are more sensitive to the factor and only the narrow pH trigger the best motility parameters. For *S*. *trutta* and *S*. *salar*, the optimum pH was established at 10, for *O*. *mykiss* at pH 9 [31,83]. For *C*. *lavaretus*, *C*. *albula*, *Salvelinus fontinalis* and *Thymallus thymallus* favorable motility were reported within the pH range 8–9 [27,28,83]. Spermatozoa of fish other than salmonids require a lower pH of 7.0–8.0 to achieve the desirable motility parameters [3,23,26,29,39,80,84–86].

The composition of seminal plasma maintains spermatozoa fish in quiescence [87]. Its composition influences spermatozoa motility and fertilization efficiency [88–90]. The osmolality of seminal plasma is isotonic or slightly higher than that of blood plasma [2,7,19,20,91]. Potassium is the only cation with a five to over a hundred times higher concentration in seminal plasma compared to blood plasma [2,7,19,20,87,91,92]. Seminal plasma properties are changed during in the course of reproductive season, depending on the age of reproductors, frequency of ejaculation, contamination during stripping hormone stimulation, time after injection and other factors [93–99]. Despite this, differences in the seminal plasma composition between taxa are noticeable [3,40,41]. In the studied *L*. *lota*, the concentration of main ions in seminal plasma were close to those for salmonids and the concentration of potassium is five times lower than that of sodium (Table 1). In the second group of fish which spermatozoa motility is controlled by osmolality of external environment potassium ions concentration in seminal plasma is more diverged. In Esocidae, the content of the ions [100,101] seem to be similar to that in salmonids, whereas in Percidae, the concentration of potassium is lower [3,10]. In the representatives of Siluriformes, the potassium level is low as well [7,92]. However, in cyprinid fishes, higher amount of potassium and lower amount of sodium were detected. The content of both ions was almost equal [2,89,93,102]. The concentration of calcium, magnesium and chloride differ slightly between species (Table 1). Acipenseriformes is a unique group with seminal plasma of lower osmolality and lower concentration of electrolytes (Table 1). Among teleosts, there are differences in the molecular mechanism leading to the activation of movement of axonemal tubules, and in the seminal plasma composition. Understanding these parameters in various taxons and their association with phylogenesis requires further studies.

The burbot is an endangered species in many parts of world. Artificial insemination and restocking of offspring is a necessity to preservation of the species [57–61]. It is known that the percentage of motile spermatozoa and spermatozoa velocity are correlated with the proportion of fertilized eggs and that spermatozoa motility characteristics are affected by external environment [40,67,103]. Determination of quality of milt, selection of the samples, maintaining the best conditions during fertilization will only allow to achieve reproductive success during artificial insemination.

### Conclusion

It was demonstrated that spermatozoa of European burbot are inhibited by K^+^ ion similarly as of North American burbot. The solution with K^+^ concentration above 12 mM (50 mOsm kg^-1^) totally ceased spermatozoa movement. Other electrolyte and non-electrolyte solutions only of osmolality far exceeding the physiological osmolality of seminal plasma (around 500 mOsm kg^-1^) inhibits spermatozoa movement. The simple solution of NaCl of osmolality 100–200 mOsm kg^-1^ and pH 9 could ensure good velocity, motility and motility duration of burbot spermatozoa.

## Supporting information

S1 Fig Database for Fig 1. **The effects of pH on European burbot spermatozoa motility at particular times after activation.** (A) percentage of motile spermatozoa (MOT). (B) duration of motility. (C) curvilinear velocity (VCL). (D) linearity (LIN). (E) amplitude of lateral head displacement (ALH). (F) beat cross frequency (BFC). Values marked with the same letter are not significantly different from one another (P>0.05). Two-way or one-way ANOVAs and then Tukey tests were used for the post-hoc comparison. Mean value ± SEM.
(XLS)

S2 Fig Database for Fig 2. **The effects of KCl concentrations on European burbot spermatozoa motility at particular times after activation.** (A) percentage of motile spermatozoa (MOT). (B) duration of motility. (C) curvilinear velocity (VCL). (D) linearity (LIN). (E) amplitude of lateral head displacement (ALH). (F) beat cross frequency (BFC). Values marked with the same letter are not significantly different from one another (P>0.05). Two-way or one-way ANOVAs and then Tukey tests were used for the post-hoc comparison. Mean value ± SEM.
(XLS)

S3 Fig Database for Fig 3. **The effects of sucrose concentrations on European burbot spermatozoa motility at particular times after activation.** (A) percentage of motile spermatozoa (MOT). (B) duration of motility. (C) curvilinear velocity (VCL). (D) linearity (LIN). (E) amplitude of lateral head displacement (ALH). (F) beat cross frequency (BFC). Values marked with the same letter are not significantly different from one another (P>0.05). Two-way or one-way ANOVAs and then Tukey tests were used for the post-hoc comparison. Mean value ± SEM.
(XLS)

S4 Fig Database for Fig 4. **The effects of NaCl concentrations on European burbot spermatozoa motility at particular times after activation.** (A) percentage of motile spermatozoa (MOT). (B) duration of motility. (C) curvilinear velocity (VCL). (D) linearity (LIN). (E) amplitude of lateral head displacement (ALH). (F) beat cross frequency (BFC). Values marked with the same letter are not significantly different from one another (P>0.05). Two-way or one-way ANOVAs and then Tukey tests were used for the post-hoc comparison. Mean value ± SEM.
(XLS)
